# Opposite changes in blood pressure and pulse rate in two patients with distigmine and rivastigmine intoxication

**DOI:** 10.1186/s40981-020-00323-w

**Published:** 2020-02-29

**Authors:** Hiraku Sedogawa, Mitsuhiro Matsuo

**Affiliations:** Department of Anesthesiology, Itoigawa General Hospital, 457-1 Takegahana, Itoigawa, Niigata, 941-8502 Japan

**Keywords:** Adverse effects, Cholinesterase inhibitors, Emergencies, Vital signs

## Abstract

**Background:**

Cholinergic crisis caused by cholinesterase inhibitors is rare but life-threatening. Clinical manifestations are thought to be similar to those caused by organophosphates.

**Case presentation:**

A 77-year-old woman on a standard dose of distigmine presented with impaired consciousness, blood pressure (BP) of 69/40 mmHg, a pulse rate (PR) of 60 beats/min, miosis, bronchorrhea, and serum cholinesterase (ChE) of 8 IU/L. After discontinuation of distigmine, altered mental status and pupil miosis were gradually resolved in 5 days with a concomitant increase of serum ChE. A 91-year-old woman presented with a headache, BP of 202/86 mmHg, PR of 83 beats/min, miosis, 9 rivastigmine patches on her knees, and ChE of 22 IU/L. The day after close observation without rivastigmine use, her symptoms were almost resolved with a concomitant increase of serum ChE.

**Conclusion:**

Our cases and a literature review suggested that, in contrast to distigmine, rivastigmine-induced cholinergic crisis caused hypertension and tachycardia.

## Background

Cholinergic crisis is an adverse drug effect associated with cholinesterase inhibitors. A nationwide database study showed that the iatrogenic cholinergic crisis is a rare but potentially life-threatening condition in Japan [1]. The clinical features resemble carbamate and organophosphate poisoning with symptoms that include both muscarinic (e.g., salivation, lacrimation, urination, defecation, miosis, bronchorrhea, and bradycardia) and nicotinic (e.g., muscle weakness, fasciculations, and paralysis) stimulation [2, 3]. Here, we present 2 cases of cholinergic crisis caused by the reversible cholinesterase inhibitors; distigmine and rivastigmine focused on changes in blood pressure (BP) and pulse rate (PR).

## Case presentation

### Case 1

A 77-year-old bedridden Japanese woman with a body mass index (BMI) of 19.8 kg/m^2^ receiving 5 mg of distigmine daily for a neurogenic bladder was transferred to the emergency department of our hospital because of impaired consciousness. On admission, she was disoriented with a Glasgow coma scale of 10 (E4V2M4), and her vital signs were BP 69/40 mmHg, PR 60 beats/min, and oxygen saturation 79% with oxygen therapy via a nonrebreather face mask at 10 L/min. Physical examination revealed pupil miosis, excess salivation, urinary incontinence, and watery diarrhea, but no obvious signs of dehydration. A blood gas analysis suggested acute hypercapnic respiratory failure (arterial carbon dioxide, 51.8 mmHg; arterial oxygen tension, 41.5 mmHg). Laboratory data showed mild leukocytosis and serum cholinesterase (ChE) of 8 IU/L. Other blood tests, including kidney function, liver function including serum albumin, C-reactive protein, and blood glucose, were within normal ranges. Her head computed tomography (CT) scan, electrocardiography, and chest X-ray were unremarkable. Her chest CT scan revealed slight centrilobular nodules with a large amount of intrabronchial secretion. She was diagnosed with distigmine-induced cholinergic crisis followed by acute hypercapnic respiratory failure because of alveolar hypoventilation due to bronchorrhea.

After admission, distigmine was discontinued, and she received supplemental oxygen therapy and ceftriaxone treatment for a possible concurrent pneumonia. Altered mental status, pupil miosis, respiratory failure, excess salivation, urinary incontinence, and watery diarrhea were gradually resolved in 5 days with a concomitant increase of serum ChE (Table 1 (A)).

**Table 1:** Changes in vital signs and serum cholinesterase before and after both patients’ admissions

	Days after admission							
(Patient A)								
	− 2 m	− 1 m	0	+ 1	+ 3	+ 12	+ 1 m	+ 2 m
BP, mmHg	100/52	110/72	69/40	104/52	105/46	110/50	104/58	130/74
PR, bpm	64	78	60	66	61	61	60	64
SpO2, %	98	98	91 (10 L)	98 (4L)	97	96	96	97
ChE, IU/L	ND	ND	8	ND	65	145	ND	ND
(Patient B)								
	− 2 m	− 1 m	0	+ 1	+ 7	+ 14	+ 1 m	+ 2 m
BP, mmHg	143/83	126/69	202/86	135/65	116/45	169/81	144/66	135/63
PR, bpm	74	75	83	85	78	83	83	69
SpO2, %	ND	ND	97	99	97	95	95	99
ChE, IU/L	ND	ND	22	78	236	ND	ND	248

Trends in vital signs and serum cholinesterase (ChE) are shown for patients A (case 1) and B (case 2). Data before admission were obtained during routine check-ups by their primary care physicians

*BP* blood pressure, *PR* pulse rate, *SpO2* oxygen saturation, *ND* not determined

### Case 2

A 91-year-old Japanese woman with Alzheimer’s disease was taken to her primary care physician for a headache and lightheadedness. The patient had normal BMI, and her nutritional status seemed to correspond to the chronological age. Her vital signs were BP 202/86 mmHg, PR 83 beats/min, and oxygen saturation 98% in room air. She was referred to our department for further evaluation. On admission to our hospital, physical examination revealed pupil miosis but no findings of lacrimation, urination, or bronchorrhea. The emergency physician noticed 9 rivastigmine patches (4 patches of a 9-mg/day formula and 5 of a 13.5-mg/day formula) attached to her both knees. She said that she mistakenly used these patches instead of topical analgesia. Laboratory data showed mild leukocytosis and a serum ChE of 22 IU/L. Other blood tests, including kidney function, liver function, C-reactive protein, and blood glucose, were within normal ranges. Her head CT scan, electrocardiography, and chest X-ray were unremarkable. She was suspected to be in cholinergic crisis caused by rivastigmine overdose and was admitted for close observation without rivastigmine use. The day after her admission, her symptoms, headache, lightheadedness, and miosis were almost resolved with a concomitant increase of serum ChE (Table 1 (B)). On day 7, her serum ChE levels returned to baseline at 236 IU/L. Consequently, she was diagnosed with rivastigmine-induced cholinergic crisis.

## Discussion

Distigmine is considered as the main causative drug of cholinergic crisis in Japan [1]. Table 2 (A) summarizes case reports describing distigmine-induced crisis. Most reports showed development of hypotension and bradycardia. In some patients, vital signs showed a circulatory shock state, requiring vasopressors. Distigmine can induce bradycardia, which is consistent with the report that pyridostigmine, a combination of two molecules in distigmine, shows heart rate reduction in patients with heart failure [26]. In contrast, as in case 2, patients with rivastigmine intoxication developed elevated BP and a relatively increased PR. These findings are compatible with previous reports (Table 2 (B)). Excessive doses of donepezil, a central acetylcholinesterase inhibitor, induces bradycardia [27–29]; therefore, cholinesterase inhibition in the central nervous system may not participate in the mechanism by which rivastigmine cause tachycardia. A standard dose of rivastigmine was shown not to be associated with any changes in BP or PR [30, 31]. Future studies are needed to elucidate the mechanisms of hyperdynamic state induced by rivastigmine overdose.

**Table 2:** Previous reports showing distigmine and rivastigmine intoxication

	BP, mmHg	PR, bpm	Age, years	Sex	Serum ChE, IU/L	Ref
(A) Distigmine	69/40	60	77	F	8	case #1
	Shock		62	M	7	[4]
	Shock		72	F	11	[5]
	Shock		76	F	11	[6]
	ND	30	77	M	ND	[7]
	50 (palpation)	40	78	M	13	[8]
	60/34	56	75	M	4	[9]
	88/50	48	70s	M	6	[10]
	93/52	32	71	M	41	[11]
	97/46	55	54	F	3	[12]
	97/64	50	40s	F	17	[13]
	100 (palpation)	56	72	M	1	[14]
	100/60	102	73	F	76	[15]
	104/60	56	73	M	16	[16]
	104/66	94	63	M	0.06ΔpH*	[17]
	119/37	39	67	F	26	[18]
	120/68	88	65	M	564 (range, 3000-6500)	[19]
	120/76	118	56	M	73	[20]
(B) Rivastigmine	202/86	83	91	F	22	case #2
	120/80	89	70	F	ND	[3]
	150/80	106	87	M	ND	[21]
	169/119	88	38	M	947 U/g hemoglobin (range, 2710-11510 U/g)	[22]
	180/90	79	59	M	6.0 μkat/L (range, 60–190 μkat/L)	[23]
	186/61	83	91	F	35	[24]
	207/85	101	80	F	ND	[25]

Vital signs and serum cholinesterase (ChE) upon initial presentation are summarized for patients A (distigmine) and B (rivastigmine)

*BP* blood pressure, *PR* pulse rate, *ND* not determined

*Reference article did not include the reference range

In case 1, a clinical dosage of distigmine had been administered for 7 years until the patient developed cholinergic crisis. Because her caregivers administered the drug, we ruled out the possibility of distigmine abuse. Her routine checkup before admission showed a stable serum creatinine level of 0.3–0.4 mg/dL over several years, suggesting a ≥ 100 mL/min/1.73m^2^ estimated glomerular filtration rate (eGFR). However, her serum creatinine was mildly elevated to 0.76 mg/dL on admission to our hospital, meaning that the eGFR decreased to 57 mL/min/1.73m^2^. Because distigmine is mainly eliminated by renal excretion [32], the relative decrease in distigmine clearance might have been associated with her distigmine overdose.

Our previous study showed an age-dependent decline in serum ChE levels in physically independent adults [33]. As shown in Table 2, a marked decrease in ChE activity was observed in all cases in which the serum ChE was measured. Lower ChE activity may be associated with lower BP and PR in patients who have experienced a distigmine overdose (Table 2 (A)). In acute organophosphate pesticide poisoning, however, associating ChE activity with disease severity remains controversial [34]. Further studies are needed to clarify whether serum ChE reflects the severity of cholinesterase-inhibitor intoxication.

## Conclusion

Our findings suggest that, in contrast to distigmine, rivastigmine-induced cholinergic crisis increases the BP and PR. Serum ChE should be periodically measured in patients with cholinesterase inhibitors to avoid life-threatening adverse effects.

## Data Availability

The datasets used during the current study are available from the corresponding author on reasonable request.

## References

[1] Ohbe H, Jo T, Matsui H, Fushimi K, Yasunaga H (2018). Cholinergic crisis caused by cholinesterase inhibitors: a retrospective nationwide database study. J Med Toxicol.

[2] Sidell FR (1994). Clinical effects of organophosphorus cholinesterase inhibitors. J Appl Toxicol.

[3] Lee Duk Hee, Choi Yoon Hee, Cho Kwang Hyun, Yun Soon Young, Lee Hyung Min (2011). A case of rivastigmine toxicity caused by transdermal patch. The American Journal of Emergency Medicine.

[4] Sakurai Toshihiro, Yamada Shu, Kitada Maki, Hashimoto Satoshi, Hashimoto Shoko, Kimura Fumihiko, Harada Masahiro, Takahashi Takeshi (2014). A case of cholinergic crisis caused by distigmine bromide administered for paralytic ileus. Journal of the Japanese Society of Intensive Care Medicine.

[5] Katayama S, Kumasawa J, Oe K, Tazawa N, Lee H, Ito C, A case of cholinergic crisis induced by chronic distigmine bromide intoxication J Jpn Soc Intensive Care Med 2011;18:227-31. (Abstract in English).

[6] Ueki M. Shirakawa Y, Aibiki M, Tukamoto I, Taie S, Umegaki O, et al. A case of distigmine bromide poisoning. Chudoku Kenkyu. 1991;4:383-5. (Abstract in English).

[7] Niinou N, Matsubara Y, Yamamori Y, Sasaki A, Ohshima Y, Kajitani H (2005). A case of cholinergic crisis associated with distigmine bromide administration. Shimane Kenritsu Chuo Byoin Igaku Zasshi..

[8] Yamanaka S, Fujita I, Murota T, Kawakita M, Matsuda T. Cholinergic crisis following administration of distigmine bromide: a case report. Hinyokika Kiyo. 2002;48:21-3. (Abstract in English). 11868380

[9] Tokimasa Y, Fujiwara K, Higo H, Kameyama N, Kayatani H, Matsuo K, et al. A case of cholinergic crisis induced by distigmine bromide successfully treated with noninvasive positive pressure ventilation. Nihon Kyobu Rinsho. 2013;72:894-9. (Abstract in English).

[10] Shinya H, Hakoda S, Kiuchi S. An aged patient with dementia of cholinergic crisis by distigmine bromide. Nihon Rinsho Kyukyu Igakkai Zasshi (Journal of Japanese Society for Emergency Medicine). 2009;19:453-7. (in Japanese).

[11] Kaneko S, Kobayashi K, Morita K, Teruya M, Kaminishi M (2016). A case of cholinergic crisis caused by distigmine bromide associated with toxic megacolon after rectal cancer resection. Geka..

[12] Kobayashi K, Sekiguchi H, Sato N, Hirose Y. Bowel obstruction-induced cholinergic crisis with progressive respiratory failure following distigmine bromide treatment. Chudoku Kenkyu. 2016;29:26-9. (Abstract in English). 27255021

[13] Nishizawa T, Matsumoto T, Nakamura T, Sasano M, Mayama Y (2019). Cholinergic crisis with cardiac arrest following distigmine bromide overdose. J Jpn Soc Intensive Care Med.

[14] Hasegawa S, Takagi T, Hirai N, Takita T (2009). A case of cerebral infarction with cholinergic crisis for term administration of distigmine bromide. Journal of Clinical Rehabilitation.

[15] Nitta K, Tsushima H, Kajihara K, Kajiwara S, Jyo H, Hara M (2016). A case of cholinergic crisis presenting with difficulties in diagnosing the cause of impaired consciousness. Hiroshima Igaku..

[16] Tada M, Fujita N. Umeda M. Koike H, Nagai H. A case of acute distigmine bromide intoxication in the therapeutic dosage for treatment of underactive neurogenic bladder. No To Shinkei. 2004;56:415-9. (Abstract in English). 15279199

[17] Iwata M, Matsui S, Maruyama M, Taniguchi H, Oda H. Miwa T, et al. A case of acute respiratory failure associated with cholinergic crisis induced by distigmine bromide. Nihon Kyobu Rinsho. 2002;61:84-91. (Abstract in English).

[18] Matsuki Y, Matsuki Y, Yasuda Y, Murakami T, Takakura K, Shigemi K, Severe bradycardia associated with cholinergic crisis induced by a small dose of distigmine bromide. Junkan Seigyo. 2013;34:78-81. (Abstract in English).

[19] Kaneko Norihiro, Kaneshige Hiroshi, Suzuki Hajime (1996). A Case of Acute Respiratory Failure with a Decrease of Serum Cholinesterase Caused by Distigmine Bromide. Nihon Shuchu Chiryo Igakukai zasshi.

[20] Morita S, Miwa H, Kondo T. A case of cholinergic crisis induced by distigmine bromide during a course of Bickerstaff encephalitis. Wakayama Igaku. 2003;54:222-4. (Abstract in English).

[21] Lövborg H, Jönsson AK, Hägg S (2012). A fatal outcome after unintentional overdosing of rivastigmine patches. Curr Drug Saf..

[22] Sener S, Ozsarac M (2006). Case of the month: rivastigmine (Exelon) toxicity with evidence of respiratory depression. Emerg Med J..

[23] Brvar M, Mozina M, Bunc M (2005). Poisoning with rivastigmine. Clin Toxicol (Phila)..

[24] Suzuki Y, Kamijo Y, Yoshizawa T, Fujita Y, Usui K, Kishino T (2017). Acute cholinergic syndrome in a patient with mild Alzheimer’s type dementia who had applied a large number of rivastigmine transdermal patches on her body. Clin Toxicol(Phila).

[25] Hoffman RS, Manini AF, Russell-Haders AL, Felberbaum M, Mercurio-Zappala M (2009). Use of pralidoxime without atropine in rivastigmine (carbamate) toxicity. Hum Exp Toxicol..

[26] Villacorta Aline Sterque, Villacorta Humberto, Caldas José Antônio, Precht Bernardo Campanário, Porto Pilar Barreto, Rodrigues Letícia Ubaldo, Neves Márcio, Xavier Analucia Rampazzo, Kanaan Salim, Mesquita Cláudio Tinoco, da Nóbrega Antônio Cláudio Lucas (2018). Effects of Heart Rate Reduction With Either Pyridostigmine or Ivabradine in Patients With Heart Failure: A Randomized, Double-Blind Study. Journal of Cardiovascular Pharmacology and Therapeutics.

[27] Pourmand A, Shay C, Redha W, Aalam A, Mazer-Amirshahi M (2017). Cholinergic symptoms and QTc prolongation following donepezil overdose. Am J Emerg Med.

[28] Shepherd Greene, Klein-Schwartz Wendy, Edwards Robin (1999). Donepezil Overdose: A Tenfold Dosing Error. Annals of Pharmacotherapy.

[29] Yano H, Fukuhara Y, Wada K, Kowa H, Nakashima K. A case of acute cholinergic adverse effects induced by donepezil overdose: a follow-up of clinical course and plasma concentration of donepezil. Rinsho Shinkeigaku. 2003;43:482-486 (in Japanese). 14658400

[30] Rosler M., Anand R., Cicin-Sain A., Gauthier S., Agid Y., Dal-Bianco P., Stahelin H. B, Hartman R., Gharabawi M., Bayer T. (1999). Efficacy and safety of rivastigmine in patients with Alzheimer's disease: international randomised controlled trial   Commentary: Another piece of the Alzheimer's jigsaw. BMJ.

[31] Isik AT, Soysal P, Yay A (2014). Which rivastigmine formula is better for heart in elderly patients with Alzheimer’s disease: oral or patch?. Am J Alzheimers Dis Other Demen.

[32] Vree TB, Waitzinger J, Hammermaier A, Radhofer-Welte S (1999). Absolute bioavailability, pharmacokinetics, renal and biliary clearance of distigmine after a single oral dose in comparison to i.v. administration of 14C-distigmine-bromide in healthy volunteers. Int J Clin Pharmacol Ther.

[33] Matsuo M, Tazawa K (2019). Reference range of clinical blood tests in physically independent patients of advanced age with groin hernia in a Japanese hospital. Geriatr Gerontol Int.

[34] Yuan Shaoxin, Gao Yusong, Ji Wenqing, Song Junshuai, Mei Xue (2018). The evaluation of acute physiology and chronic health evaluation II score, poisoning severity score, sequential organ failure assessment score combine with lactate to assess the prognosis of the patients with acute organophosphate pesticide poisoning. Medicine.

